# Ag_8_SnS_6_: a new IR solar absorber material with a near optimal bandgap†

**DOI:** 10.1039/c8ra08734b

**Published:** 2018-11-26

**Authors:** Patsorn Boon-on, Belete Asefa Aragaw, Chun-Yen Lee, Jen-Bin Shi, Ming-Way Lee

**Affiliations:** Institute of Nanoscience, Department of Physics, National Chung Hsing University Taichung 402 Taiwan mwl@phys.nchu.edu.tw; Department of Chemistry, Bahir Dar University P.O. Box 79 Bahir Dar Ethiopia; Department of Electronic Engineering, Feng Chia University Taichung 40724 Taiwan

## Abstract

We report the synthesis and photovoltaic properties of a new ternary solar absorber – Ag_8_SnS_6_ nanocrystals prepared by successive ionic layer adsorption reaction (SILAR) technique. The synthesized Ag_8_SnS_6_ nanocrystals have a bandgap *E*_g_ of 1.24–1.41 eV as revealed from UV-Vis and external quantum efficiency (EQE) measurements. Its photovoltaic properties were characterized by assembling a liquid-junction Ag_8_SnS_6_ sensitized solar cell for the first time. The best cell yielded a *J*_sc_ of 9.29 mA cm^−2^, a *V*_oc_ of 0.23 V, an FF of 31.3% and a power conversion efficiency (PCE) of 0.64% under 100% incident light illumination using polysulfide electrolyte and Au counter electrode. The efficiency improved to 1.43% at a reduced light intensity of 10% sun. When the polysulfide was replaced by a cobalt electrolyte with a lower redox level, the *V*_oc_ increased to 0.54 V and PCE increased to 2.29% under 0.1 sun, a respectable efficiency for a new solar material. The EQE spectrum covers the spectral range of 300–1000 nm with a maximum EQE of 77% at *λ* = 600 nm. The near optimal *E*_g_ and the respectable photovoltaic performance suggest that Ag_8_SnS_6_ nanocrystals have potential to be an efficient IR solar absorber.

## Abstract 2

We report the synthesis and photovoltaic properties of a new ternary solar absorber – Ag_8_SnS_6_ nanocrystals prepared by successive ionic layer adsorption reaction (SILAR) technique.

## Graphical abstract

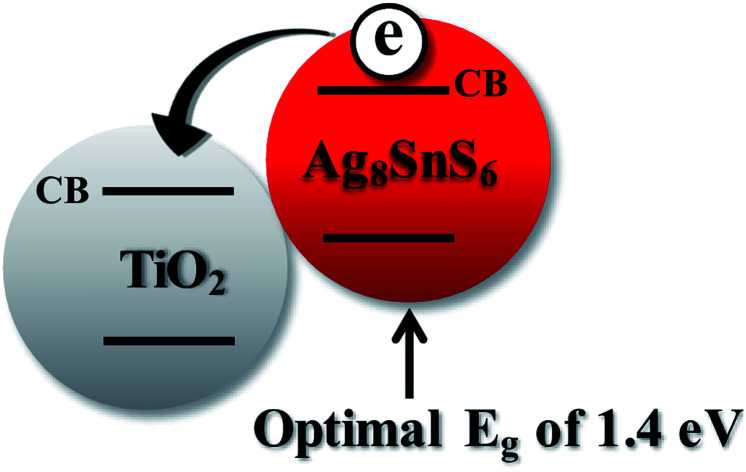

## 1. Introduction

Semiconductor nanocrystals are now receiving much research attention in the field of photovoltaics. This is due to the unique properties of semiconductor NC materials such as band gap tenability^1,2^ and high absorption coefficient.^3^ Moreover, multiple exciton generation effect and hot electron injection in these solar absorbers could improve the solar cell efficiency beyond the Shockley–Queisser limit.^4–9^ Semiconductor nanocrystals materials can be prepared by solution-based processing, which has the advantages of ease of fabrication and low-cost over Si based solar cell processing.

A requirement for a good solar absorber material is that the energy gap *E*_g_ should be near 1.4 eV in order to produce a maximal output power.^10^ To date, the most widely studied solar absorber materials have been the binary metal sulfides and selenides such as CdS, CdSe, PbS, PbSe, Sb_2_S_3_ and Ag_2_S *etc.*^11–16^ For a binary semiconductor, its *E*_g_ is a fixed value. Only a small number of binary semiconductor satisfies the *E*_g_ = 1.4 eV requirement. This limits the number of binary semiconductors suitable for solar absorbers. An advantage of ternary semiconductors is that *E*_g_ can be tuned by varying the ratios of the constituent elements, leading to formation of a broad range of new compounds that could be potential solar absorber materials. Solar cells based on ternary metal sulfides have been relatively less explored due to the difficulty in the material synthesis (the widely studied Cu–In–Se system being an exception).

The family of I–III/IV–VI (Cu- and Ag-based) ternary metal sulfides, such as AgInS_2_,^17^ AgBiS_2_,^18^ AgSbS_2_,^19^ Ag_3_SbS_3_,^20^ CuInS_2_,^16^ Cu_2_SnS_3_ (ref. 21 and 22) and Cu_4_SnS_4_,^23,24^ have been investigated for their applications in photovoltaics and photocatalytics. Among them, Ag_8_SnS_6_ owns several important properties such as an ideal *E*_g_ of 1.3–1.5 eV, which is near the optimal *E*_g_ of 1.4 eV for a solar cell, and high absorption coefficients of *α* ∼ 10^4^ cm^−1^ in the visible range.^25^ Moreover, the three elements contained in Ag_8_SnS_6_ – Ag, Sn and S – are nontoxic and environmentally friendly. Ag_8_SnS_6_ nanocrystals have been synthesized for various purposes^26–30^ such as counter electrodes in dye-sensitized solar cells,^31^ photocatalytic dye degradation,^32^ photoelectrochemical salt-water splitting^33^ and thermoelectrics.^34^ Ag_8_SnS_6_ solar cells have recently been reported with an efficiency of 0.25%.^35^ Here, we report the simple solution-based preparation of Ag_8_SnS_6_ nanocrystals and investigation of their photovoltaic properties. Ag_8_SnS_6_ nanocrystals were directly grown on the surfaces of mesoporous TiO_2_ nanoparticles using the successive ionic layer adsorption and reaction (SILAR) technique. Liquid-junction quantum dot-sensitized solar cells based on Ag_8_SnS_6_ nanocrystals are demonstrated for the first time. The material crystal structure, morphology and optical property have been investigated. The dependence of photovoltaic performance on SILAR conditions, sun intensity and type of electrolyte have also been studied.

## 2. Experimental

### 2.1. Preparation of TiO_2_ electrodes

Fluorine-doped tin oxide glass (FTO, Pilkington, sheet resistance ∼ 7 Ω □^−1^) was cleaned with acetone, methanol, and deionized water successively in an ultrasonic bath. It was then coated with three layers of TiO_2_ film with different particle sizes and film thicknesses. First, a 90 nm thick compact TiO_2_ film was made by spin-coating (2000 rpm, 1 min) a 0.2 M titanium tetraisopropoxide solution (TTIP) onto an FTO substrate, followed by annealing at 190 °C for 5 min. Next, a mesoporous TiO_2_ (mp-TiO_2_) layer (Dyesol 30NR-D, particle size 30 nm, ∼10 μm thick) was coated on top of the compact layer by the doctor blade technique and heated at 125 °C for 5 min. Finally, a TiO_2_ scattering layer (Dyesol WER2-O, particle size 300 nm, ∼5 μm thick) was coated on top of the mp-TiO_2_ layer and heated at 500 °C for 15 min.

### 2.2. SILAR growth of Ag_8_SnS_6_ nanocrystals

The formation of Ag–Sn–S nanocrystals was made by a two-stage SILAR process. In the first stage, Ag–S nanoparticles were grown on the mp-TiO_2_ electrode. In the second stage, Sn–S nanoparticles were grown on top of the Ag–S nanoparticles. Post-annealing transformed the Ag–S/Sn–S double-layered structure into the Ag–Sn–S phase. An Ag–S SILAR cycle was performed by dipping the TiO_2_ electrode in the solution of AgNO_3_ (0.1 M) for 30 s accompanied with rinsing and drying the electrode. The step resulted in the adsorption of Ag ions on the pores of mp-TiO_2_. The electrode was then dipped in a Na_2_S (0.1 M) solution. This process is called one SILAR cycle and creates the Ag_2_S nuclei. Repetition of this process is necessary to increase the particle size of Ag_2_S nuclei. In the second-stage SILAR cycle for Sn–S, the same procedure was followed except changing the AgNO_3_ cation precursor solution with SnCl_2_ solution to make Sn–S. To achieve high performance, the numbers of Ag_2_S and Sn–S SILAR cycles need to be optimized. The best ratio for Ag_2_S and Sn–S SILAR cycles were found to be 3 : 2. For example, a sample with 12 Ag_2_S cycles has 8 Sn–S cycles. To simplify discussion, a sample with Ag_2_S (12 cycles)/Sn–S (8 cycles) will be referred to as sample with SILAR(12) herein. Finally, the Ag_2_S/Sn–S deposited mp-TiO_2_ electrode was annealed at 400 °C for 10 min under flowing N_2_ gas, which resulted in the formation of ternary phase Ag_8_SnS_6_ nanoparticles.

### 2.3. Fabrication of solar cells

The solar cells were fabricated by a sandwich-type assembly of the Ag_8_SnS_6_ nanocrystal-immobilized TiO_2_ photoelectrode and counter electrode (CE) using a 190 μm-thick parafilm thermoplastic spacer and sealant. Gold (Au) was used as the CE. A polysulfide electrolyte containing 0.5 M Na_2_S, 2 M S, 0.2 M KCl and 0.5 M NaOH in a methanol/DI water (volume ratio 7 : 3) solution was used as a redox mediator. The cobalt electrolyte consisted of 0.2 M Co^2+^ (Co[PyPz]_3_[PF_6_]_2_ salt-Dyesol), 0.05 M Co^3+^ (Co[PyPz]_3_[PF_6_]_2_-Dyesol), 0.2 M LiClO_4_, and 0.2 M *t*-butyl pyridine with acetonitrile solution, where Py is pyridine and Pz is pyrazole. The electrolyte was injected separately through a predrilled hole on the counter electrode into the cell. The Au CE film, ∼70 nm in thickness, was deposited onto an FTO substrate by sputtering evaporation.

### 2.4. Material characterization and photovoltaic measurements

The crystal structure and phase purity of the prepared material were studied using a high-resolution X-ray diffractometer (XRD, Bruker D8 SSS). The particle size and morphology were determined by a transmission electron microscope (TEM, Joel JEM-2010). Optical properties were studied using a Hitachi U-2800A UV-Vis spectrophotometer. The photovoltaic performance was studied by measuring photocurrent–voltage (*I*–*V*) curves using a Keithley 2400 source meter under 100 mW cm^−2^ light illumination from an Oriel 150 W Xe lamp with an Oriel bandpass filter simulating the AM 1.5 solar spectrum. External quantum efficiency (EQE) was measured using the monochromatic light generated from an Acton monochromator with a 250 W tungsten halogen lamp. The active area, defined by a metal mask, was 3 mm × 3 mm.

## 3. Results and discussion

### 3.1. Morphology and crystal structure characterization

**Fig. 1:**
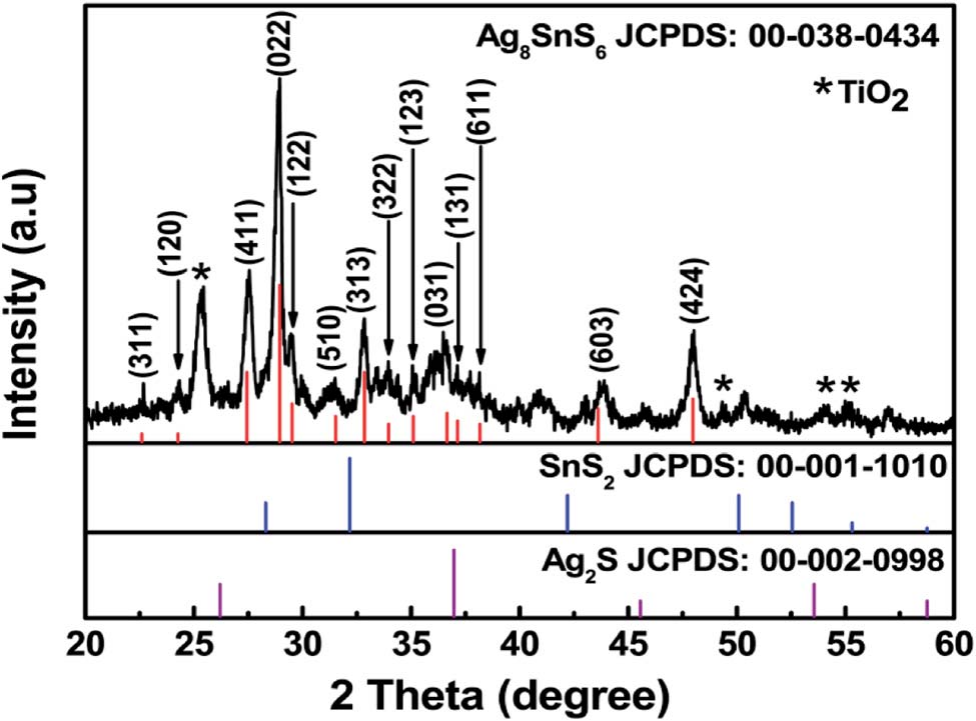
XRD spectra of Ag_8_SnS_6_ nanocrystals along with the reference JCPDS 00-038-0434, reference patterns of SnS_2_ and reference pattern of Ag_2_S.

The structural property of the prepared Ag–Sn–S ternary phase was investigated by studying its X-ray diffraction (XRD) pattern after annealing at 400 °C under nitrogen atmosphere. The XRD pattern shown in Fig. 1 indicates the formation of orthorhombic Ag_8_SnS_6_ structure that is in agreement with the reference JCPDS no. 00-038-0434. The diffraction planes given at (311), (120), (411), (022), (122), (510), (313), (322), (123), (031), (611), (131), (603) and (424) are characteristic of the orthorhombic Ag_8_SnS_6_ structure. The (022) peak shows the strongest peak intensity compared with other peaks signifying the preferential orientation or major growth of the crystal along (022) plane. The other peaks due to the TiO_2_ and FTO substrates were also observed. The calculated lattice constants for orthorhombic Ag_8_SnS_6_ are *a* = 15.29, *b* = 7.57 and *c* = 10.71 Å, which are in good agreement with the lattice constants of the data in the JCPDS database. For comparison, the major peaks associated with the binary Ag_2_S and SnS_2_ compounds are displayed in the bottom panel. It is clear the Ag_8_SnS_6_ XRD pattern does not contain the two starting binary phases, indicating successful formation of the Ag_8_SnS_6_ single phase. The deposited Ag–S/Sn–S double-layered particles are mostly amorphous before heat treatment (see Fig. S1, ESI†). Only a weak Ag_2_S peak at 37° corresponding to the (200) plane can be observed. The average particle size of Ag_8_SnS_6_ nanocrystals, calculated by the Scherrer's formula^36^ given in eqn (1) using the strongest (022) peak, is about 17.4 nm.1
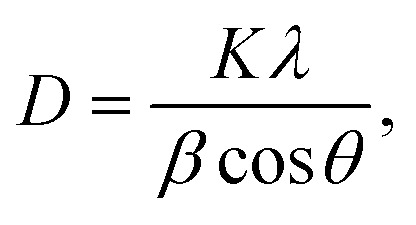
where *D* is the average particle size, *λ* is the wavelength of the incident X-rays, *β* is the value of the full width at half maximum, *K* is a numerical constant and *θ* is the Bragg angle.

**Fig. 2:**
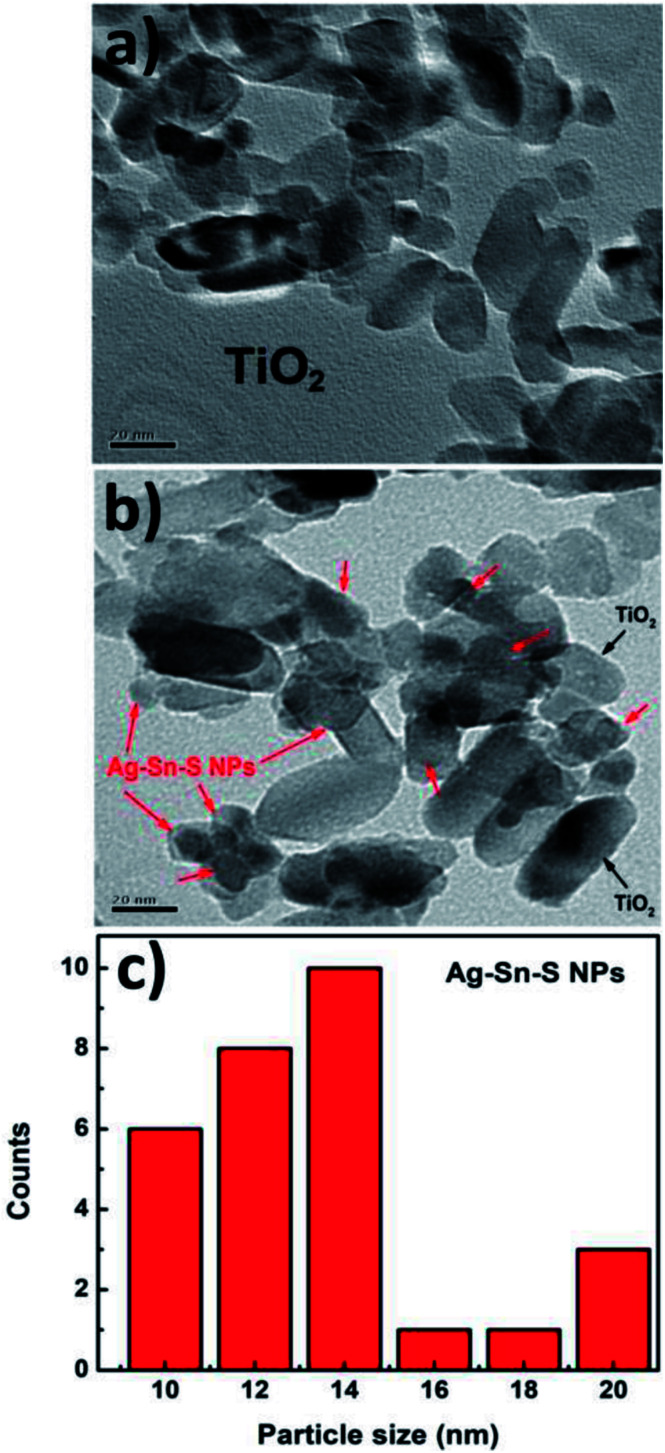
TEM images of (a) 30 nm bare TiO_2_ nanoparticles, (b) Ag_8_SnS_6_ nanocrystals deposited on TiO_2_ nanoparticles and (c) size distribution of Ag_8_SnS_6_ nanocrystals.

Fig. 2a displays a TEM image of bare TiO_2_ nanoparticles. The rectangular shaped TiO_2_ particles have round corners and an average length of 30 nm. Fig. 2b displays a TEM image of Ag_8_SnS_6_ nanocrystals grown on the surface of TiO_2_ nanoparticles. The Ag_8_SnS_6_ nanocrystals, marked by red arrows, are randomly distributed all over the pores of mesoporous TiO_2_ with no visible aggregation. Fig. 2c shows the size distribution of Ag_8_SnS_6_ nanocrystals prepared with eight SILAR cycles. The particle size of Ag_8_SnS_6_ nanocrystals is the range of ∼10–20 nm with a distribution peak near 15 nm. This size is in agreement with the value (17 nm) calculated using Scherrer's formula.

### 3.2. Optical property

**Fig. 3:**
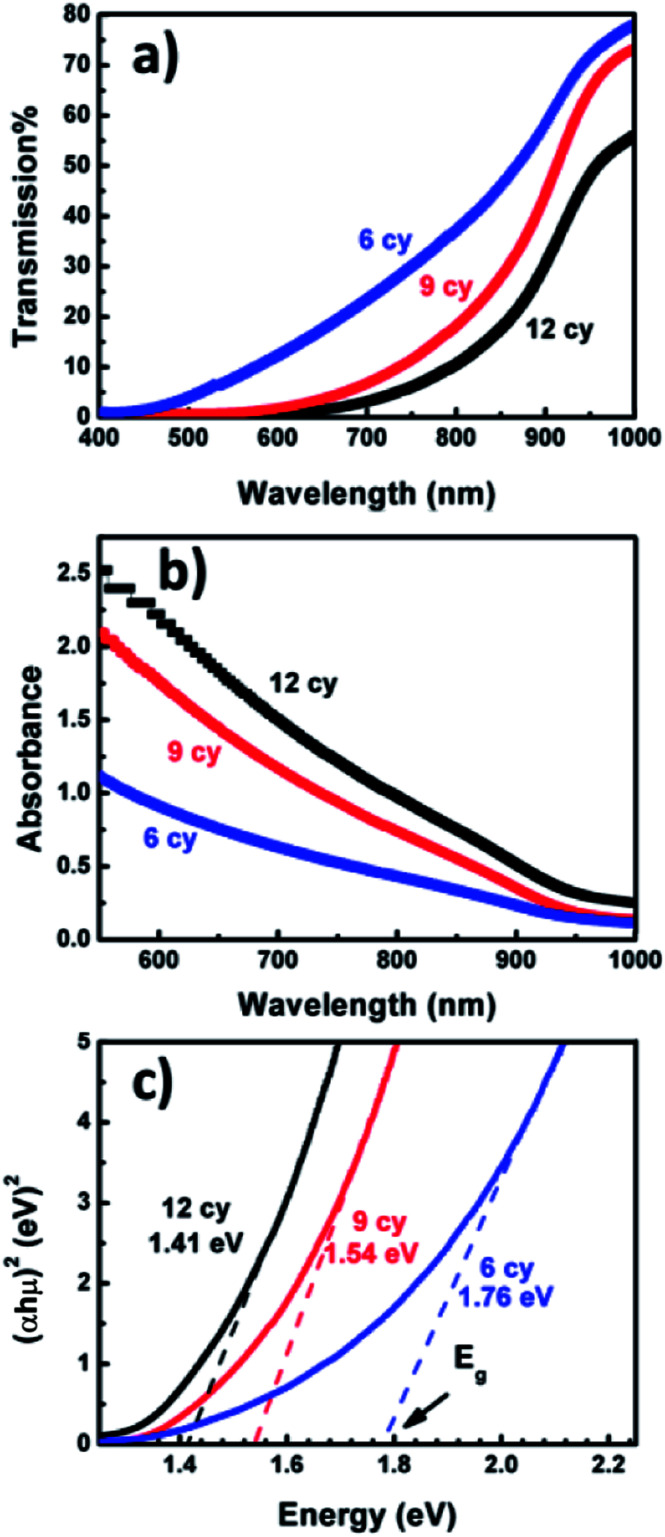
Optical spectra (a) transmission, (b) absorbance and (c) Tauc plots (*αhν*)^2^*versus hν*.

Fig. 3a shows the UV-Visible transmission spectra *T*(*λ*) of Ag_8_SnS_6_ nanocrystals with different numbers of SILAR cycles *n*. The corresponding optical absorbance *A*(*λ*), calculated from the optical transmission value using the relation 
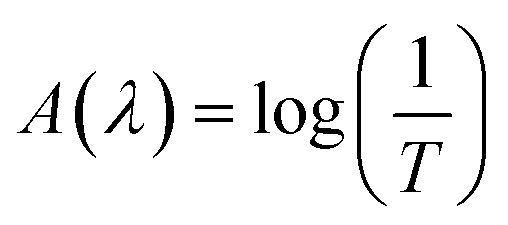
, is displayed in Fig. 3b. The transmission *T*(*λ*) decreases with increasing *n*, indicating increasing light absorption by the increased amount of material with increasing SILAR cycles. The absorbance *A*(*λ*) increases with increasing *n*, which is, again, the result of increasing material. A notable feature of the transmission spectra is that the small transmission (*T*(*λ*) ≤ 5%, *n* = 12 sample) for wavelengths < 700 nm. This implies the Ag_8_SnS_6_ nanocrystals can absorb nearly all the photon energy with wavelength lower than 700 nm (the visible and near IR regions of the solar spectrum). The large light absorption ability is an advantageous property for a solar material.

Fig. 3c shows the Tauc plot, (*αhν*)^2^*versus hν*, of the Ag_8_SnS_6_ nanocrystals where *h* is the Planck constant and *ν* is the frequency. The optical band gap *E*_g_ can be estimated by finding the x-intercept of an extrapolated Tauc plot. *E*_g_ decreases with increasing SILAR cycles *n* as: 1.76, 1.54, 1.41 eV for SILAR cycles of 6, 9, 12, respectively. The decrease in *E*_g_ with increasing *n* is attributed partly to the quantum size effect – a large SILAR cycle produced larger particles and, hence, a lower *E*_g_. The *E*_g_ observed here is in agreement with the literature values for Ag_8_SnS_6_ nanocrystals prepared by other synthesis methods (1.31–1.45 eV).^31,33,35^ Moreover, The *E*_g_ of 1.41 eV (*n* = 12 sample) is equal to the optimal *E*_g_ (1.4 eV) for a solar absorber, which is favorable property for a solar material.

### 3.3. Photovoltaic performance

**Fig. 4:**
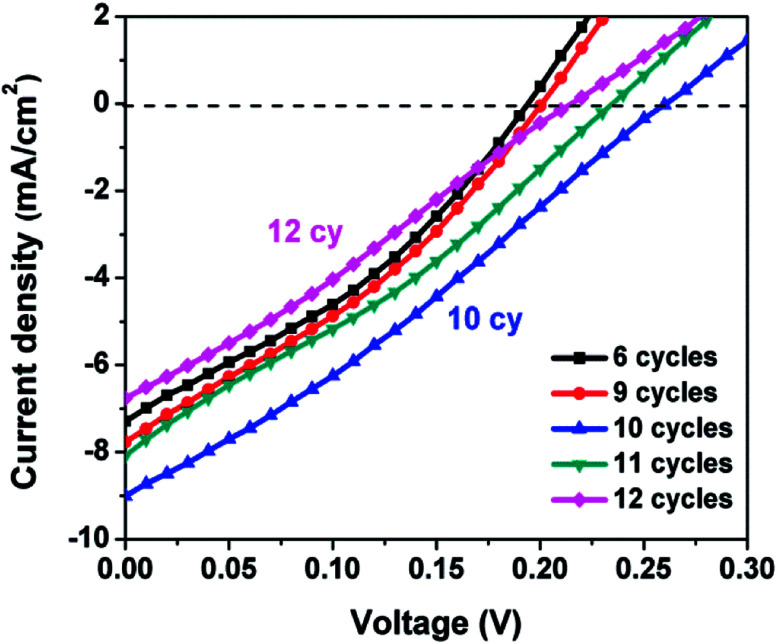
*J*–*V* curves of Ag_8_SnS_6_ QDSSCs with various numbers of SILAR cycles *n*.

The photovoltaic performance of a SILAR-prepared QDSSC is strongly dependent on the number of SILAR cycles *n*. An insufficient or excess amount of semiconductor absorber material leads to low efficiencies. Fig. 4 displays the *J*–*V* curves for Ag_8_SnS_6_ QDSSCs with various numbers of SILAR cycles *n*. The CE was Au and the electrolyte was polysulfide. Table 1 lists the photovoltaic parameters – short-circuit current density (*J*_sc_), fill factor (FF), open-circuit voltage (*V*_oc_) and power conversion efficiency (PCE). The efficiency increased with SILAR cycles *n*, reaching a maximal PCE of 0.68% at *n* = 10 (sample no. 3), then decreased again for a larger SILAR cycle of *n* = 11 (sample no. 4). The optimal sample (no. 3) has the photovoltaic parameters of *J*_sc_ = 9.01 mA cm^−2^, open-circuit voltage *V*_oc_ = 0.26 V and FF = 28.9%. These are typical results for QDSSCs prepared by SILAR. Initially, the amount of material deposited on the mp-TiO_2_ electrode was low, leading to insufficient solar light harvesting and low efficiencies. The deposited material increased with the SILAR cycle, leading to improved efficiencies. This result had been observed in CdS and other semiconductor solar cells as reported previously.^37,38^ After reaching the optimal SILAR cycle of 10, a further increase in SILAR cycle led to over-saturated material among the porous spaces within the mp-TiO_2_ matrix, which hindered the flow of liquid electrolyte and led to a reduced efficiency.

**Table 1:** Photovoltaic performance of Ag_8_SnS_6_ quantum dot-sensitized solar cells with various numbers of SILAR cycles. Electrolyte: polysulfide. Light intensity: 1 sun. Counter electrode: Au

Sample no.	SILAR cycle	*J*_sc_ (mA cm^−2^)	*V* (V)	FF (%)	PCE (%)
1	6	7.29	0.19	34.07	0.47
2	9	7.77	0.20	32.45	0.50
3	10	9.01	0.26	28.90	0.68
4	11	8.08	0.23	30.25	0.56
5	12	7.62	0.24	29.91	0.55

**Fig. 5:**
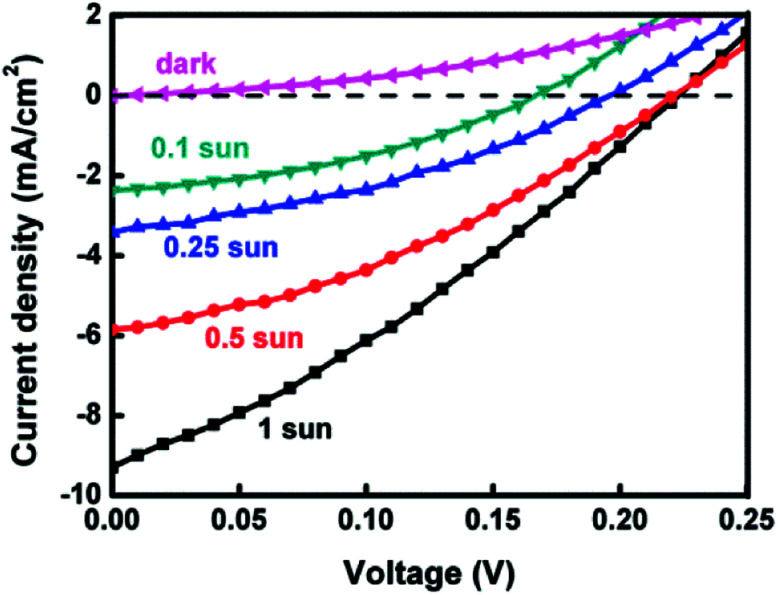
*J*–*V* curves of Ag_8_SnS_6_ QDSSCs under various light intensities. Electrolyte: polysulfide.

The photovoltaic performance of a QDSSC could be improved by measuring *J*–*V* curves under reduced light intensities. Fig. 5 displays the *J*–*V* curves of the best Ag_8_SnS_6_ cell under various light intensities. The cell had an Au CE and a polysulfide electrolyte. Table 2 lists the photovoltaic parameters. As the incident light intensity was reduced from 100% sun to 10% sun, the PCE increased from 0.64% to 1.43%, a significant increase of 123%. The improved PCE is due to (a) an increase in FF from 31.3% to 39.7%, an improvement of 27%; and (b) an improvement in *J*_sc_ due to the reduction in carrier recombination (explained below). For an ideal solar cell, the current density *J*_sc_ should be linearly proportional to the number of incident photon *n*_ph_. As the light intensity was reduced from 1 to 0.1 sun, the number of incident photons *n*_ph_ was also reduced to 0.1*n*_ph_. Hence, the linear response model predicted that *J*_sc_ at 0.1 sun should equal to 9.29 mA cm^−2^ (1 sun) × 0.1 = 0.929 mA cm^−2^. However, the experimental data in Table 2 shows a much higher *J*_sc_ of 2.25 mA cm^−2^ under 0.1 sun. Semiconductor nanocrystals prepared by SILAR inherently contain a large number of surface defects, which act as recombination sites for photocarriers. The mechanism of recombination can be revealed by analyzing the dependence of *J*_sc_ on light intensity *I*_0_. Analysis of the data in Table 2 yielded a sublinear relation: *J*_sc_ ∝ *I*_0_^α^ where *α* = 0.67. Nelson has explained the sublinear *J*_sc_–*I*_0_ relation in terms of the multiple trapping model for carrier recombination.^39^ A solar cell with multiple trapping would experience reduced carrier recombination at low light intensities and yield improved performance. The most important effect of low light intensities is the increase in electron lifetime *τ*_n_ under low light. Miyashita *et al.* and A. C. Fisher *et al.* had observed in dye-sensitized solar cells that *τ*_n_ increased by ∼3–10 times as the light intensity was decreased from 100 to 10% sun.^40,41^ The significantly improved performance under low light intensities indicates that carrier recombination is the limiting process in the liquid-junction Ag_8_SnS_6_ sensitized solar cell at high incident light intensities. The competition between recombination and extraction of free charges determines FF, *J*_sc_ and inturn the PCE of the cell. At low light intensities, the charge carrier recombination process is reduced, which improves the charge extraction process and the performance of the solar cell.^42^

**Table 2:** Photovoltaic performance of Ag_8_SnS_6_ quantum dot-sensitized solar cells under various sun intensities

Sun intensity	*J*_sc_ (mA cm^−2^)	*V*_oc_ (V)	FF (%)	*η* (%)
100%	9.29	0.23	31.3	0.64
50%	5.85	0.22	35.4	0.92
25%	3.42	0.19	36.6	0.96
10%	2.25	0.16	39.7	1.43

Reducing light intensities also has an effect on *V*_oc_. As seen in Table 2, *V*_oc_ decreased monotonically with light intensity. As the light intensity decreased from 100% to 10% sun, *V*_oc_ decreased from 0.23 to 0.16 V. This can be explained as follows. The theoretical maximal *V*_oc_ of a QDSSC is the difference between the quasi Fermi level *E*_F_ of TiO_2_ and the redox potential *E*_redox_ of the electrolyte, *i.e.*,

2 *V*_oc_ = *E*_F_ − *E*_redox_.

The Fermi level *E*_F_ depends on the electron density *n*_CB_ in the conduction band (CB) of TiO_2_ according to:^43^*E*_F_ = *k*_B_*T* ln(*n*_CB_)

A reduced light intensity generates a reduced number of photoelectrons *n*_CB_, leading to a lower *E*_F_ and *V*_oc_ as shown in Table 2.

**Fig. 6:**
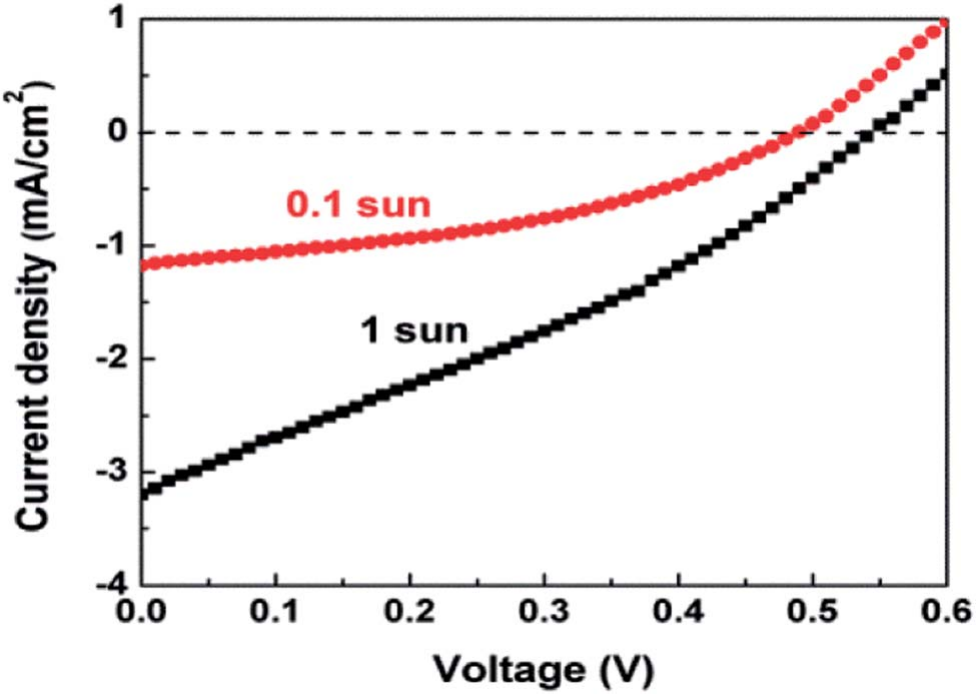
*J*–*V* curves of an Ag_8_SnS_6_ QDSSC employing cobalt electrolyte under 1 and 0.1 sun.

A disadvantage of the Ag_8_SnS_6_ QDSSC is that the *V*_oc_ had a low value of 0.24 V, which lowered the attainable efficiency. One way to improve *V*_oc_ is to use a different electrolyte. As eqn (2) indicates, a lower redox potential *E*_redox_ will produce a higher *V*_oc_. Here the polysulfide electrolyte in the Ag_8_SnS_6_ QDSSC was replaced by the cobalt electrolyte. Cobalt electrolytes have a redox level of 0.86 V relative to NHE (normal hydrogen electrode) whereas the polysulfide electrolyte has a redox level of −0.77 V.^44^ The difference is 1.57 V. The cobalt electrolyte produced a much larger *V*_oc_ of 0.54 V (Table 3) under 1 sun, as compared to 0.24 V of the polysulfide electrolyte. The PCE of the cobalt-electrolyte Ag_8_SnS_6_ QDSSC is 0.53% under 1 sun. However, at the reduced light intensity of 0.1 sun, the PCE greatly increased to 2.29% (Fig. 6), which is 60% higher than that (1.43%) of the polysulfide cell.

**Table 3:** Photovoltaic performance of Ag_8_SnS_6_ quantum dot-sensitized solar cells using a cobalt electrolyte

Light intensity	*J*_sc_ (mA cm^−2^)	*V*_oc_ (V)	FF (%)	PCE (%)
1 sun	3.20	0.54	30.61	0.528
0.1 sun	1.18	0.48	40.60	2.29

A known disadvantage of cobalt electrolytes is its low diffusion coefficient and slow kinetics for hole transfer in electrolytes, resulting a low *J*_sc_. So the cobalt electrolyte works better under low light intensities when the small number of photoelectrons is small. A cobalt electrolyte will produce a low *J*_sc_ and a high *V*_oc_. The large gains in *V*_oc_ and FF compensate loss in *J*_sc_ and increase the PCE to 2.29% under 0.1 sun.

**Fig. 7:**
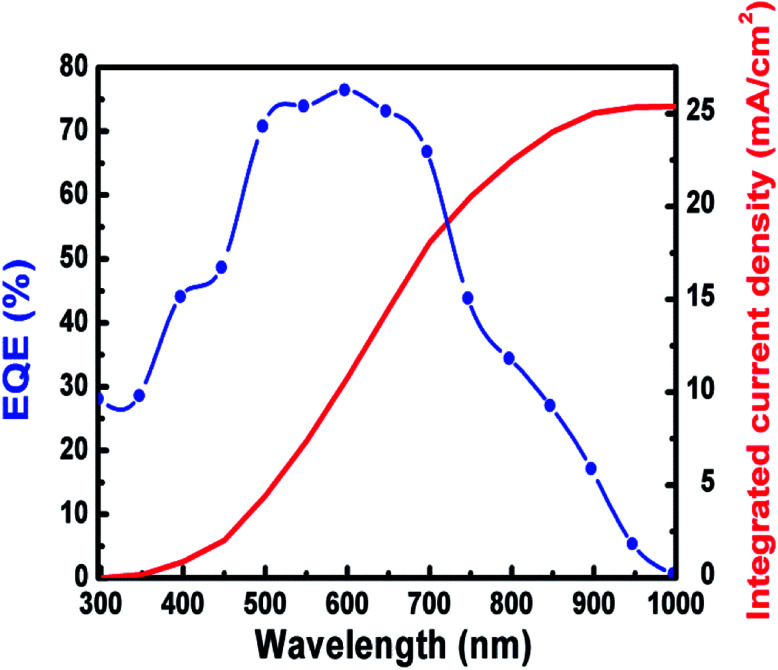
EQE spectrum.

Fig. 7 displays the EQE spectrum for the best Ag_8_SnS_6_ QDSSCs in the 300–1000 nm wavelength range, where a maximum EQE value of 77% at *λ* = 600 nm was achieved. The cell started to produce a current at the onset wavelength of 1000 nm. The *E*_g_ of Ag_8_SnS_6_ nanocrystals, calculated from the onset wavelength of the EQE spectrum using the relation: 
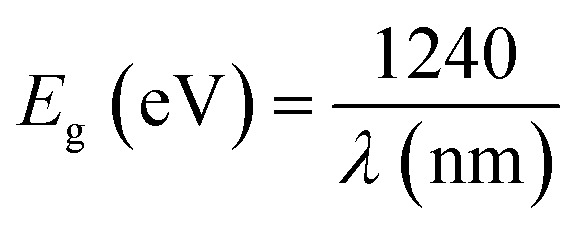
, is 1.24 eV, which is close to the optical *E*_g_ value of 1.41 eV shown in Fig. 4. Therefore, we conclude that the Ag_8_SnS_6_ nanocrystal has an *E*_g_ value of 1.24–1.41 eV. This *E*_g_ equals to the optimal *E*_g_ (1.4 eV) for a solar absorber, showing its potential as a solar absorber material. The area under the EQE curve represents the total integrated current density *J*_ph_ generated from the solar cell. *J*_ph_ can be calculated from the EQE curve using the following equation:
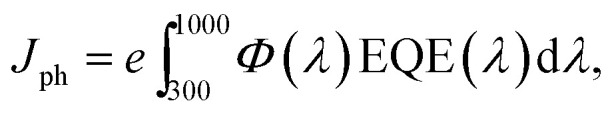
where *Φ*(*λ*) is the incident photon flux and *e* is the elementary charge. The integrated EQE resulted in a maximum *J*_ph_ of 25.5 mA cm^−2^ (shown in the right axis of Fig. 7). The EQE measurement is basically equivalent to a low-light *I*–*V* measurement because the single-wavelength light dispersed from a monochromator has a relatively low power intensity. Compared to Table 2, the integrated *J*_ph_ of 25.5 mA cm^−2^ is consistent with the *J*_sc_ of 2.25 mA cm^−2^ under 0.1 sun (*i.e.* 25.5 × 0.1 sun = 2.55 mA cm^−2^).

## 4. Conclusion

We demonstrated liquid-junction Ag_8_SnS_6_ QDSSCs prepared by SILAR on a mp-TiO_2_ electrode. The orthorhombic Ag_8_SnS_6_ nanocrystals have an *E*_g_ of 1.24–1.41 eV and an average size of 15 nm. The cell employing polysulfide electrolyte exhibited a *V*_oc_ of 0.23 V, and a PCE of 1.43% at 0.1 sun. In contrast, the cell employing cobalt electrolyte yielded a higher PCE of 2.29% at 0. 1 sun. The near optimal *E*_g_ and the broad absorption band suggest that Ag_8_SnS_6_ nanocrystal could be a promising candidate material for solar cells.

## Conflicts of interest

There are no conflicts to declare.

## Supplementary Material

RA-008-C8RA08734B-s001
