# Supplementary material for: Ag8SnS6: a new IR solar absorber material with a near optimal bandgap
Source: RSC Adv. 2018 Nov 26;8(69):39470–6. doi: 10.1039/c8ra08734b (PMC9091033; doi:10.1039/c8ra08734b)
Supplement: RA-008-C8RA08734B-s001 [file RA-008-C8RA08734B-s001.pdf]

Electronic Supplementary Material (ESI) for RSC Advances.
This journal is © The Royal Society of Chemistry 2018

Supplementary Information

Ag_8SnS_6: a new IR Solar Absorber Material with a near Optimal Bandgap

Patsorn Boon-on,[a] Belete Asefa Aragaw[a,b], Chun-Yen Lee,[a] Jen-Bin Shi[c] and Ming-Way Lee[a,1]

[a]*Institute of Nanoscience and Department of Physics, National Chung Hsing University, Taichung 402, Taiwan*

[b]*Department of Chemistry, Bahir Dar University, P.O. Box 79, Bahir Dar, Ethiopia*

[c]*Department of Electronic Engineering, Feng Chia University, Taichung, 40724, Taiwan*

[1] Corresponding author e-mail: mwl@phys.nchu.edu.tw

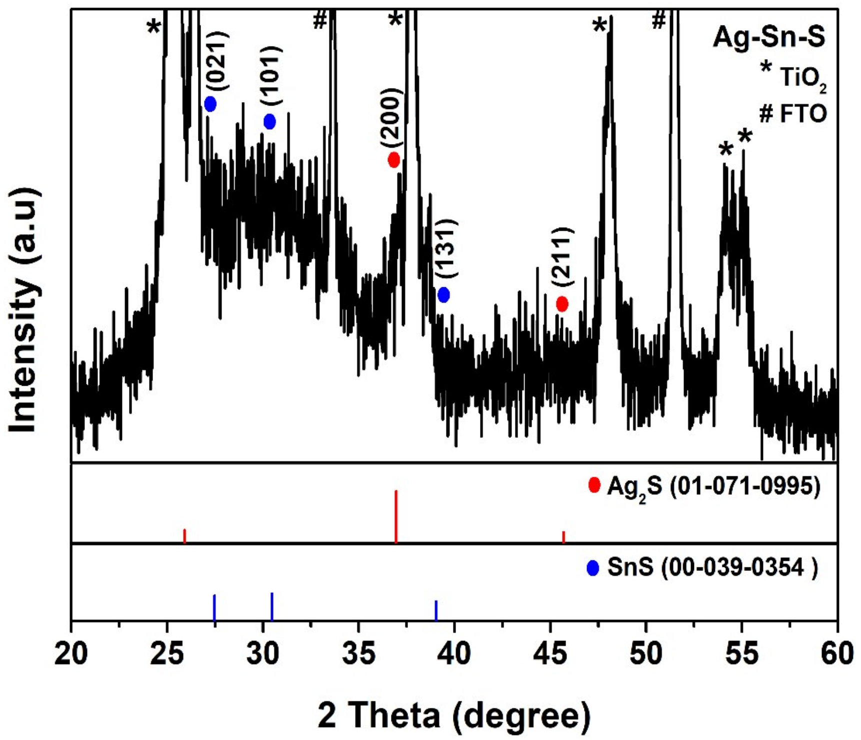

Fig. S1 XRD pattern of Ag-Sn-S sample before heat treatment. The material is mostly amorphous. Only a weak Ag_2S peak at 37° corresponding to the (200) plane is observed.
